# A technique for improving petroleum products forecasts using grey convolution models and genetic algorithms

**DOI:** 10.1016/j.mex.2023.102097

**Published:** 2023-02-27

**Authors:** Flavian Emmanuel Sapnken, Ahmat Khazali Acyl, Michel Boukar, Serge Luc Biobiongono Nyobe, Jean Gaston Tamba

**Affiliations:** a Laboratory of Technologies and Applied Science, University Institute of Technology, University of Douala, PO Box 8698, Douala, Cameroon; b Transports and Applied Logistics Laboratory, University Institute of Technology, University of Douala, PO Box 8698, Douala, Cameroon

**Keywords:** Energy demand, Optimization, Arc consistency, Constraint satisfaction problems, Sequential-GMC(1,n)-GA hybrid model, *Sequential-GMC(1,n)-GA hybrid model*

## Abstract

Forecasting energy consumption is a major concern for policymakers, oil industry companies, and many other associated businesses. Though there exist many forecasting tool, selecting the most appropriate one is critical. GM(1,1) has proven to be one of the most successful forecasting tool. GM(1,1) does not require any specific information and can be adapted to predict energy consumption using a minimum of four observations. Unfortunately, GM(1,1) on its own will generate too large forecast errors because it performs well only when data follow an exponential trend and should be implemented in a political-socio-economic free environment. To reduce these short-comings, this paper proposes a new GM(1,n) convolution model optimized by genetic algorithms integrating a sequential selection mechanism and arc consistency, abbreviated Sequential-GMC(1,n)-GA. The new model, like some recent hybrid versions, is robust and reliable, with MAPE of 1.44%, and RMSE of 0.833.

• Modification, extension and optimization of grey multivariate model is done.
• The model is very generic can be applied to a wide variety of energy sectors.
• The new hybrid model is a valid forecasting tool that can be used to track the growth of households’ energy demand.

## Graphical abstract

**Figure:**
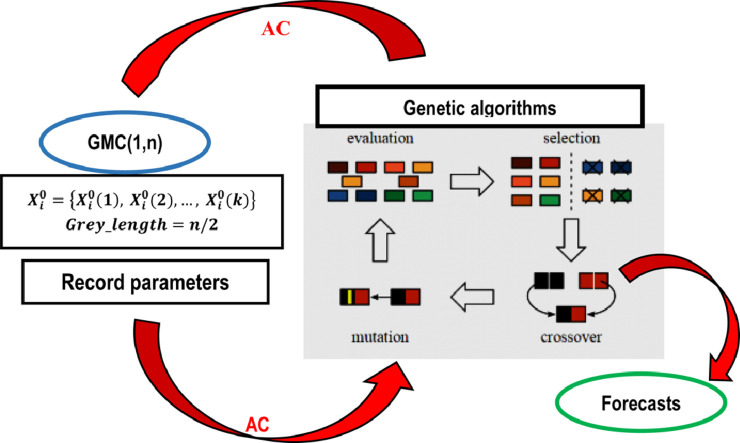

Specifications table

**Table:** 

Subject area	Energy
More specific subject area	*Modeling and forecasting*
Name of your method	*Sequential-GMC(1,n)-GA hybrid model*
Name and reference of original method	*T.-L. Tien, A research on the grey prediction model GM(1,n), Applied Mathematics and Computation. 218 (2012) 4903–4916.* *B. Bogaerts, E. Gamba, T. Guns, A framework for step-wise explaining how to solve constraint satisfaction problems, Artificial Intelligence. 300 (2021) 103550. https://doi.org/10.1016/j.artint.2021.103550.* *J. Holland, Adaptation in Natural and Artificial Systems, Second Edition, University of Michigan Press, 1975.*
Resource availability	*World Bank statistics (https://data.worldbank.org)* *Hydrocarbon Price Stabilization Fund (https://www.csph.cm)* *Petroleum Products data (https://www.scdp.cm)* *National Institute of Statistics (https://www.minefop.gov.cm)*

## Introduction

In 2017, the household sector was Cameroon's largest energy consumer, accounting for 70% of overall energy consumption, thereby over distancing transports (15%) and industries (11%) [1]. It was also the fastest growing sector over the period 2001–2010, going from 1942.78 kilotons of oil equivalent (ktoe) to 3995.57 ktoe, although strongly dominated by biomass with a share of 94.74% (3785.6 ktoe), followed by petroleum products (PP) (125.13 ktoe, 3.13%) and electricity (84.84 ktoe, 2.12%) [1]. In the following years, this trend is projected to continue, due to major urbanization projects initiated by the State of Cameroon. Cameroon thus finds itself torn between two major challenges: ensuring the development of its household sector without ignoring climatic issues. To achieve these projects, the certainty on energy consumption prediction is quite important.

### Interest of study

The legal obligation imposed by regulatory authorities is one reason for conducting forecasting studies in Cameroon's oil market. Besides, in cases where distributors cause an imbalance in the supply chain of PPs, related regulations and laws impose a high penalty payment on them [2]. This has the effect of putting pressure on distributors to accurately forecast end-users’ consumption.

Forecast studies are also important because PPs supply is provided through spot markets, where the bulk of the demand is in urban areas. Most households in Cameroon consume the bare minimum of their PP needs, as the latter are only used for lighting (kerosene) and/or cooking (kerosene and liquefied petroleum gas (LPG)) [2]. It is therefore incumbent on the State, and according to the market's structure, to manage its own strategic stocks in order to regulate PP's consumption.

### Related works

There exist many forecasting techniques [3], however, genetic algorithms (GA) and grey models (GM) are different from all others by their simplicity. In addition, they can be applied with a minimum of four data points. Unfortunately, GMs alone are not sufficient to forecast energy demand because, very often, forecast results are not sufficiently precise and sometimes, too strong assumptions are made which could irremediably lead to unacceptable forecast errors [4]. GA have gained the attention, as they are robust stochastic search algorithms that are used to solve various problems, including forecasting. GA have several considerable advantages over conventional methods. They can provide practical solutions, by analysing the search space from many starting points, without making any prior assumption about the model or the underlying function [5].

Amongst existing grey models, GM(1,1) has had success in many fields including energy, earthquakes, finance, food production, education, road safety and transports. However, GM(1,1) works well when data follow an exponential trend and this is only applicable in a political-socio-economic free environment. Yet, such a neutral environment does not reflect the reality of a generalized grey system. This is why data collected from such an environment cannot be modelled with GM(1,1) because there would be incomplete information. To complete this information, more variables must be inserted into the system. This tends to suggest that a multivariate grey model (GM(1,n)) is a way forward to whiten the system. Unfortunately, as demonstrated by Tien [5], GM(1,n) and GM(m,n) all lead to inaccurate modelling because of their structural defects.

The traditional GM(1,n) can be improved by adjusting its structure to make it more flexible either by directly optimizing and stabilizing its parameters, and by considering the hysteresis of the related variables [6]. Other lines of thought include new accumulation generating operation, hybridization, or by integrating a functional mechanism into the GM's structure. To date, no work has been carried out on the integration of specific mechanisms into the forecasting framework of GM(1,n). This could take into consideration key drivers of energy consumption, by connecting GM with GA.

This method proposes a sequential grey multivariable convolution model optimized by GA (Sequential-GMC(1,n)-GA). The technical innovation here is to integrate the sequential selection mechanisms in GMC(1,n) and the arc-consistency (AC) in GA as a filter with the aim of reducing CPU execution time. In this perspective, GMC(1,n) is trained by GA in order to enhance all parameters and improve prediction accuracy. The motivation of combining GMC(1,n) with GA are threefold, namely:

To whiten the grey system under consideration rendering its information completely extractable. When this is done, the hybrid model can then correctly capture predictable structure in the demand history including: trend; seasonality; and special events (e.g. peaks) that could impact demand.
To optimize all GM parameters rather than using theoretical values. This ensures that all possible solutions have been explored and tested, given way to unbiased predictions, meaning that the estimated forecast is not projecting too high or too low.
To gather the most recent data characteristics which minimizes predicting errors, CPU execution time, and the amount of retracing required because inescapable faults may be quickly identified.

As novelty, this method proposes a GMC that fully excavates the evolution law of multivariate time series without modifying the model's framework. Sequential-GMC(1,n)-GA proactively improves all model parameters in each projected time-frame, regardless of data patterns. Thus, Sequential-GMC(1,n)-GA eliminates the inconsistency issue between parameterization approach that is based on grey derivative and the model's minimal sum of squares of prediction errors, resulting in more accurate forecasts. Finally, the model is capable of using input data with different sizes and still compete with performant forecasting models.

Section 2 that follows presents the method. Section 3 presents the application using real data. Setup for model's evaluation is given in Section 4, while Section 5 is the conclusion.

## Method

### Convolution grey prediction model

GMC(1,n) model is based on six steps [5]:

Step 1 Constructing input sequences

Let $\left(X_{1}^{\left(0\right)},\;X_{2}^{\left(0\right)},\ldots ,\;X_{n}^{\left(0\right)}\right)$ be variables of a grey system. $\left(X_{1}^{\left(0\right)},\;X_{2}^{\left(0\right)},\ldots ,\;X_{n}^{\left(0\right)}\right)$ are used to construct the input sequence. Each variable in the input sequence is defined by Eq. (1):

(1) $$X_{i}^{\left(0\right)}=\left\{x_{i}^{\left(0\right)}\left(1\right),\;x_{i}^{\left(0\right)}\left(2\right),\ldots ,\;x_{i}^{\left(0\right)}\left(k\right)\right\};\;i=1,\;2,\ldots ,n;\;k\geq 4$$

$X_{i}^{\left(0\right)}$ is a nonnegative sequence; k is the sample number of the ith input variable, and the superscript (0) denotes the original sequences. $X_{1}^{\left(0\right)}$ is the output and $X_{i}^{\left(0\right)},\;i=2,\;3,\ldots ,\;n$ are the variables.

Step 2 Accumulated Generating Operation (AGO)

When $X_{i}^{\left(0\right)}$ is subject to AGO, the following Eq. (2) is obtained:

(2) $$X_{i}^{\left(1\right)}=\left\{x_{i}^{\left(1\right)}\left(1\right),\;x_{i}^{\left(1\right)}\left(2\right),\ldots ,\;x_{i}^{\left(1\right)}\left(k\right)\right\}$$

where:

(3) $$x_{i}^{\left(1\right)}\left(t\right)=\sum_{m=1}^{t}x_{i}^{\left(0\right)}\left(m\right);\;t=2,\;3,\ldots ,\;k$$

The superscript (1) in Eq. (3) represents the first order AGO of the original sequences. $x_{i}^{\left(1\right)}\left(k\right)$ increases continuously. The sequence is therefore monotonous.

Step 3 Designing the background value of the system

The system's background value is defined as in Eq. (4):

(4) $$Z_{1}^{\left(1\right)}\left(t\right)=\theta X_{1}^{\left(1\right)}\left(t\right)+\left(1-\theta \right)X_{1}^{\left(1\right)}\left(1-t\right);\;t=2,\;3,\ldots ,\;T$$

The horizontal adjustment coefficient θ is taken such that 0<θ<1 [7]. The value of θ is generally taken as 0.5 but it should be chosen so as to reduce the forecast errors [7].

Step 4 Establishing Grey systems of equations

Grey system is trained by establishing a link between known and unknown sequences:

(5) $$dx_{1}^{\left(1\right)}\left(t\right)/dt+ax_{1}^{\left(1\right)}\left(t\right)=b_{2}x_{2}^{\left(1\right)}\left(t\right)+b_{3}x_{3}^{\left(1\right)}\left(t\right)+\cdots +b_{n}x_{n}^{\left(1\right)}\left(t\right)+u$$

a is the development coefficient, u is GMC(1,n) parameter, while $b_{j=1,\ldots ,\;n}$ are grey input coefficients. Tien [5] starts by considering the right hand side of Eq. (5) as a function f(t). Then, using the trapezoid formula, and integrating both sides of Eq. (5) from t−1 to t, Eq. (5) is approximated by the following difference equation:

(6) $$x_{1}^{\left(0\right)}\left(t\right)+az_{1}^{\left(1\right)}\left(t\right)=b_{2}z_{2}^{\left(1\right)}\left(t\right)+b_{3}z_{3}^{\left(1\right)}\left(t\right)+\cdots +b_{n}z_{n}^{\left(1\right)}\left(t\right)+u$$

As a result, Eq. (6) can be regarded as a linear equation system in relation to the coefficients ${\left[a\;b_{2}\;b_{3}\ldots \;b_{n}\;u\right]}^{T}$. These coefficients are calculated by least squares method. Thus, by applying least squares, ${\left[a\;b_{2}\;b_{3}\ldots \;b_{n}\;u\right]}^{T}$ are determined as shown in Eq. (7):

(7) $$A=\left(\begin{array}{c} \begin{array}{c} a\\ b_{2}\\ b_{3} \end{array}\\ \begin{array}{c} \vdots \\ \begin{array}{c} b_{n}\\ u \end{array} \end{array} \end{array}\right)={\left(B^{T}B\right)}^{-1}B^{T}Y$$

Where: $B=\left(\begin{array}{c} \begin{array}{c} -z_{1}^{\left(1\right)}\left(2\right)\\ -z_{1}^{\left(1\right)}\left(3\right)\\ \vdots \end{array}\\ -z_{1}^{\left(1\right)}\left(n\right) \end{array}\;\begin{array}{c} \begin{array}{c} x_{2}^{\left(1\right)}\left(2\right)\\ x_{2}^{\left(1\right)}\left(3\right)\\ \vdots \end{array}\\ x_{2}^{\left(1\right)}\left(n\right) \end{array}\;\begin{array}{c} \begin{array}{c} x_{3}^{\left(1\right)}\left(2\right)\\ x_{3}^{\left(1\right)}\left(3\right)\\ \vdots \end{array}\\ x_{3}^{\left(1\right)}\left(n\right) \end{array}\;\begin{array}{c} \begin{array}{c} \cdots \\ \cdots \\ \ddots \end{array}\\ \cdots \end{array}\;\begin{array}{c} \begin{array}{c} x_{n}^{\left(1\right)}\left(2\right)\\ x_{n}^{\left(1\right)}\left(3\right)\\ \vdots \end{array}\\ x_{n}^{\left(1\right)}\left(n\right) \end{array}\right)$; $Y=\left(\begin{array}{c} \begin{array}{c} x_{1}^{\left(0\right)}\left(2\right)\\ x_{1}^{\left(0\right)}\left(3\right)\\ \vdots \end{array}\\ x_{1}^{\left(0\right)}\left(n\right) \end{array}\right)$

Step 5 Solving the system's differential equation

The solution to Eq. (5) is [5]:

(8) $${\hat{x}}_{1}^{\left(1\right)}\left(t\right)=x_{1}^{\left(0\right)}\left(1\right)e^{a\left(1-t\right)}+\int_{1}^{t}e^{a\left(\tau -t\right)}f\left(\tau \right)d\tau ;\;t\geq 2$$

Whose value can only be approximated with numerical methods because of the presence of convolution integral. With trapezoid formula, Eq. (8) becomes:

(9) $${\hat{x}}_{1}^{\left(1\right)}\left(t\right)=x_{1}^{\left(0\right)}\left(1\right)e^{a\left(1-t\right)}+0.5h\left(t\right)\sum_{i=2}^{n}\left[f\left(\tau \right)e^{a\left(\tau -t\right)}+f\left(\tau -1\right)e^{a\left(\tau -t-1\right)}\right];\;t\geq 2$$

Recall that ${\hat{x}}_{1}^{\left(1\right)}\left(1\right)=x_{1}^{\left(0\right)}\left(1\right)$, and h(t) in Eq. (9) is defined as: h(t)=0 if t<2; else h(t)=1

Step 6 Inverse AGO (IAGO)

Finally, forecasted values of ${\hat{x}}_{1}^{\left(0\right)}\left(t\right)$ are obtained by IAGO as shown in Eq. (10):

(10) $${\hat{x}}_{1}^{\left(0\right)}\left(t\right)={\hat{x}}_{1}^{\left(1\right)}\left(t\right)-{\hat{x}}_{1}^{\left(1\right)}\left(t-1\right);\;t\geq 2$$

### Optimizing GMC(1,n) with GA using arc consistency (AC) technique

The parameters k,θ,a and $b_{j=1,\ldots ,\;n}$ affect the precision of GMs. In this method, the best values of these parameters are determined experimentally (instead of using arbitrary values reported in the literature).

#### Formalism of constraint satisfaction problems (CSPs)

A CSP [8] P is a quadruplet P=(χ,D,C,R), where:

• χ represents a finite set of N variables $\left\{k,\theta ,b_{j=1,\ldots ,\;n}\right\}$.
• D denotes a set of N domains $\left\{D_{1},\;...,\;D_{N}\right\}$. Each domain $D_{i}$ is the finite set of values for the variable $\chi_{i}$.
• C represents a finite set of m constraints $\left\{C_{1},\;...,\;C_{m}\right\}$. Each constraint $C_{p}$ is defined by the set of variables $Var\left(C_{p}\right)\;=\;\left\{\chi_{P_{1}},\;...,\;\chi_{PN_{P}}\right\}\subseteq \chi$.
• R denotes a set of m relations $\left\{R_{1},\;...,\;R_{m}\right\}$. Each relation $R_{P}$ is a subset of the cartesian product $D_{P_{1}}\times D_{P_{2}}\times \;...\;\times D_{PN_{P}}$.

The arity of a constraint is the number of variables on which the constraint relates. A CSP with arity larger than 2 is an N-ary CSP. A solution is the assignment of a value to all variables of the problem such that each constraint is satisfied. CSP is consistent if and only if it admits a solution. A tuple t of the constraint $C_{p}$ is said to be allowed if and only if $t\;\in \;R_{P}$ and said to be supported for a value $v\;\in \;D_{i},\;\chi_{i}\in Var\left(C_{p}\right)$, if and only if t is allowed and t contains v in the position corresponding to $\chi_{i}$ in the constraint. Checking whether a given tuple is allowed by a constraint is called consistency testing. The possible combinations of variable instantiations in a CSP create a search space that can be seen as a search tree. We used AC algorithms to non-binary CSPs with nAC3 N-ary algorithm based on the principle of AC3 [8].

For constraint checking, we used the more popular nFC3 forward search algorithm. When instantiating a variable, nFC3 removes from the current domains of future variables all values incompatible with this instantiation. When a new variable is considered, we can then be sure that all the values of its current domain are consistent with the past variables. The computational steps of nAC3 N-ary algorithm based on the principle of AC3 is given below:

CSP Algorithm

**Table:** 

Start
Step 1: Create a variable set $\chi =\{\chi_{1},\chi_{2},\ldots ,\chi_{N}\}$
$\chi =\{k,\theta ,b_{j=1,\ldots ,\;n}\}$
Where:
$\left\{ \begin{array}{c} \;\chi_{1}=k;\;\chi_{2}=\theta \;\\ \;\chi_{3}=b_{1};\ldots ;\chi_{N}=b_{n} \end{array}\right.$
Step 2: Create a domain set $D=\{D_{1},\;...,\;D_{N}\}$
Where:
$\left\{ \begin{array}{c} D_{1}=D_{\left(k\right)};\;D_{2}=D_{\left(\theta \right)}\;\\ D_{3}=D_{\left(b_{1}\right)};\ldots ;D_{N}=D_{\left(b_{n}\right)} \end{array}\right.$
Step 3: Create a constraint set with χ and D after considering the constraints C
$C=\{C_{1},\;...,\;C_{m}\}$
Where:
$\left\{ \begin{array}{c} \begin{array}{c} \;C_{1}:4\leq k<\frac{n}{2}\;\\ C_{2}:0<\theta <1\; \end{array}\\ C_{3}:b_{1}\neq b_{2}\neq \ldots \neq b_{n}\; \end{array}\right.$
Step 4: Do while $\left(C_{i}:true,\;\forall i\right)$ and $\left(D_{i}:true,\;\forall i\right)$
$AC\left(C_{\chi_{i},\chi_{j}}\right)=\forall \chi_{i}\in D_{\left(\chi_{i}\right)},\;\exists \chi_{j}\in D_{\left(\chi_{j}\right)},\;C_{\chi_{i},\chi_{j}}\left(\chi_{i},\chi_{j}\right)$
End while
Output $\chi =\{k,\theta ,b_{j=1,\ldots ,\;n}\}$
End

#### Genetic algorithms

We consider a population made of n individuals and we want to optimize m=3+n parameters, i.e. k,θ,a and $b_{j=1,\ldots ,\;n}$. So, each chromosome will have m genes. The population P(t) is given by Eq. (11):

(11) $$P\left(t\right)=\left\{ \begin{array}{c} I_{1}=\left(k_{1}^{\left(1\right)},\theta_{2}^{\left(1\right)},a_{3}^{\left(1\right)};b_{4}^{\left(1\right)};b_{5}^{\left(1\right)};\ldots ;b_{m}^{\left(1\right)}\right)\\ I_{2}=\left(k_{1}^{\left(2\right)},\theta_{2}^{\left(2\right)},a_{3}^{\left(2\right)};b_{4}^{\left(2\right)};b_{5}^{\left(2\right)};\ldots ;b_{m}^{\left(2\right)}\right)\\ \cdots \cdots \cdots \cdots \cdots \cdots \cdots \cdots \cdots \cdots \cdots \cdots \cdots \\ I_{N}=\left(k_{1}^{\left(N\right)},\theta_{2}^{\left(N\right)},a_{3}^{\left(N\right)};b_{4}^{\left(N\right)};b_{5}^{\left(N\right)};\ldots ;b_{m}^{\left(N\right)}\right) \end{array}\right.$$

Binary forms of k,θ,a and $b_{j=1,\ldots ,\;n}$ are obtained from Eq. (12).

(12) $$k,\theta ,a\;\text{or}\;b_{j}=L+\frac{\alpha }{2^{\beta }-1}\left(U-L\right)$$

where α is the number in decimal form which is represented in binary form, β is the number of bits, L and U are the lower and upper threshold values respectively. Selection, crossover and mutation operators are then used (over a series of generations) by a standard GA to guide P(t) towards convergence at the global optimum [9].

Once selection, reproduction and mutation operators have been applied, new phenotypes (population) are created (which theoretically has a better forecast accuracy). The generational counter is incremented by one. The whole process is reiterated until $g_{max}$ is reached or until the desired forecast accuracy is achieved.

Unluckily, GA are well-known for being CPU-intensive (they sometimes take a long time when executing a given problem). To tackle this issue, we assumed that each individual in P(t) is a CSP that we are attempting to solve using AC, with P(t=1) serving as the outcome. Further specifics are in [10].

#### Sequential GMC(1,n) prediction model optimized by GA

k,θ,a and $b_{j=1,\ldots ,\;n}$ are found in the literature to be 0.5 and 4 for θ and k respectively, while a and $b_{j=1,\ldots ,\;n}$ are calculated only once during the modelling stage. However, periodic values of these parameters can significantly increase accuracy of GMC(1,n) [5]. Hence, instead of using values reported in the literature, we rather calculate them experimentally (using AC-GA) for each forecast period h.

Sequential mechanism makes use of the most up-to-date simulations for prediction by depicting the latest characteristics of data. This allows the sequential mechanism to improve forecasting accuracy. This mechanism employs p sequences to model GMC(1,n), and q consumption data for forecasting. At each loop, new values of k,θ,a, $b_{j=1,\ldots ,\;N}$ are computed and optimized by GA. The process is described below:

i. Construct GMC(1,n) using $\left[X_{i}^{\left(0\right)}\left(1\right),\;X_{i}^{\left(0\right)}\left(2\right),\ldots ,\;X_{i}^{\left(0\right)}\left(p\right)\right]$ and compute $k,\theta ,a,b_{j=1,\ldots ,\;n}$
ii. Optimize $k,\theta ,a,b_{j=1,\ldots ,\;n}$ with AC-GA and forecast $\left[{\hat{X}}_{1}^{\left(0\right)}\left(p+1\right),\;{\hat{X}}_{1}^{\left(0\right)}\left(p+2\right),\ldots ,\;{\hat{X}}_{i}^{\left(0\right)}\left(p+q\right)\right]$.
iii. Remove $\left[X_{i}^{\left(0\right)}\left(1\right),\;X_{i}^{\left(0\right)}\left(2\right),\ldots ,\;X_{i}^{\left(0\right)}\left(p\right)\right]$ from the sequence, reconstruct GMC(1,n) with the most up-to-date p sequences, i.e. $\left[X_{i}^{\left(0\right)}\left(q+1\right),\;X_{i}^{\left(0\right)}\left(q+2\right),\ldots ,\;X_{i}^{\left(0\right)}\left(q+p\right)\right]$ and compute new values of $k,\theta ,a,b_{j=1,\ldots ,\;n}$.
iv. Use AC-GA to optimize newly computed $k,\theta ,a,b_{j=1,\ldots ,\;n}$, and forecast $\left[{\hat{X}}_{1}^{\left(0\right)}\left(p+q+1\right),\;{\hat{X}}_{1}^{\left(0\right)}\left(p+q+2\right),\ldots ,\;{\hat{X}}_{i}^{\left(0\right)}\left(p+2q\right)\right]$.
v. Repeat steps (ii) to (iv) till all required consumption data are forecasted.

**Fig. 1:**
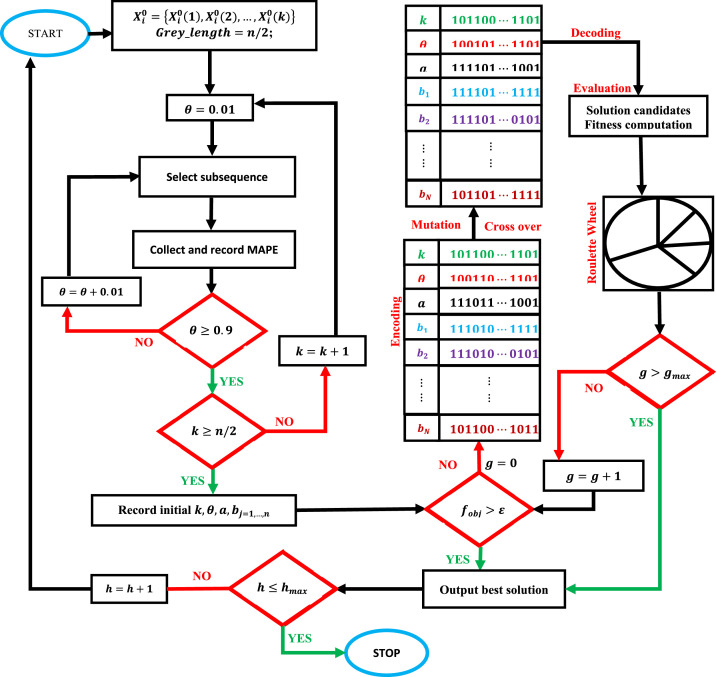
Flow chart for the proposed Sequential-GMC(1,n)-GA.

This process is called sequential GMC(1,n) prediction optimized by GA (sequential-GMC(1,n)-GA) and is summarized in Fig. 1. The above-mentioned instructions have been written with the AC (steps ii and iv). It is therefore a Sequential-GMC(1,n)-GA with AC. By editing the instruction "with AC" in steps ii and iv, it becomes a Sequential-GMC(1,n)-GA without AC.

## Application of Sequential-GMC(1,n)-GA using real data

Useful variables must be separated from the unnecessary ones. Stepwise regression analysis (SRA) defines predictor variables that most accurately characterize the variable to be predicted [11]. For this, we start by regressing the dependant variable on a single independent variable and set the significance level and values of $F_{r}$ (F-to-remove) and $F_{e}$ (F-to-enter); F being the Fisher statistic. The statistical significance of the F-test and the decrease in the sum of the squared error constitute the benchmark for adding or eliminating a variable. After inserting a new variable in the regression model, the partial F-value is calculated and compared to $F_{e}$ and $F_{r}$; if $F>F_{e}$, then we include this variable; otherwise, if $F<F_{r}$, it is eliminated and will never return to the model again. All useful variables used for this work are in annual frequency and are presented in Table 1.

**Table 1:** Inputs used in this study.

	Variable	Start	End
$X_{1}$	Petroleum products consumption	1994	2017
$X_{2}$	Lag 1 of petroleum products consumption	1993	2016
$X_{3}$	Lag 2 of petroleum products consumption	1992	2015
$X_{4}$	Price of petroleum products	1992	2017
$X_{5}$	Real income	1994	2014
$X_{6}$	Number of households	2000	2014
$X_{7}$	Urbanization rate	1998	2014

The modelling period was selected for 1992–2008, while the test period is 2009–2017. Dataset is divided into simulation (or training) set and validation (or test) set to ensure that the model is neither overfitting nor underfitting. Training dataset spans from 1992 to 2008 while validation dataset is from 2009 to 2017.

Prices and historical consumption were collected from HPSF (https://www.csph.cm) and confirmed by CCPD data (https://www.scdp.cm). Urbanization rate and number of households were provided by the National Institute of Statistics (https://www.minefop.gov.cm), while real income was collected from the World Bank statistics (https://data.worldbank.org).

### Performance measurements

Threshold levels for MAPE, r and $R_{adj}^{2}$ are given in Table 2. For RMSE and AAE, low values ​​close to 0 indicate the best predictions [12]. The speed of convergence and stability, i.e. quality and reliability, are used to assess GA's performance. The best solution obtained in relation to the number of cycles (computational cost) is used to determine speed of convergence, while the standard deviation of the results from 10 optimization runs per algorithm is used to determine stability.

**Table 2:** Accuracy measures and threshold levels.

Criteria	Formula	Threshold levels			
		1st (Perfect)	2nd (Good)	3rd (Acceptable)	4th (Poor)
MAPE‡	$\frac{1}{N}\sum_{t=1}^{N}\left|\frac{X_{1}\left(t\right)-{\hat{X}}_{1}\left(t\right)}{X_{1}\left(t\right)}\right|\times \;100$	≤0.01	≤0.05	≤0.1	>0.1
r*	$\frac{\frac{1}{N-1}\sum_{t=1}^{N}\left(X_{1}\left(t\right)-{\bar{X}}_{1}\right)\left({\hat{X}}_{1}\left(t\right)-{\bar{\hat{X}}}_{1}\right)}{\sqrt{sd{\left(X_{1}\right)}^{2}sd{\left({\hat{X}}_{1}\right)}^{2}}}$	≥0.98	≥0.95	≥0.90	<0.90
$R_{adj}^{2}$⁎⁎	$1-\frac{N-1}{N-p-1}\left(1-\frac{\sum_{t=1}^{n}{\left(X_{1}\left(t\right)-{\hat{X}}_{1}\left(t\right)\right)}^{2}}{\sum_{t=1}^{n}{\left(X_{1}\left(t\right)-{\bar{X}}_{1}\left(t\right)\right)}^{2}}\right)$	≥0.98	≥0.95	≥0.90	<0.90
RMSE†	$\sqrt{\frac{1}{N}\sum_{t=1}^{N}{\left(X_{1}\left(t\right)-{\hat{X}}_{1}\left(t\right)\right)}^{2}}$				
AAE††	$\frac{1}{N}\sum_{t=1}^{N}\left|X_{1}\left(t\right)-{\hat{X}}_{1}\left(t\right)\right|$				

‡ mean absolute percentage error,

⁎ coefficient of correlation,

⁎⁎ adjusted r.

† root mean squared error,.

†† average absolute error.

## Setup for model's evaluation

Sequential-GMC(1,n)-GA is initialized with parameters as indicated in Table 3. Once approximate values of $k,\theta ,a,b_{j=1,\ldots ,\;n}$ are obtained, they are encoded into binary digits and integrated into GA. Amongst these parameters, mutation probability must be very low, as low as 0.05 or even smaller. Because a higher value could destroy the solution. $P_{m}$ is set as the inverse of chromosome length. Crossover probability $P_{c}$ depends on the problem at hand. Its optimal value is estimated after different runs based on the targeted precision ε. If optimal values of $P_{m}$ and $P_{c}$ are settled, then number of generations and population size are simply set arbitrarily. However, a population size of 100 and 50 generations is advised.

**Table 3:** Initialization parameters.

Approach	Parameter	Value
GMC(1,n)	horizontal adjustment coefficient, θ	0.01
	k	4
	Number of variables, n	7
	Grey length	12
Approach	Parameter	Value
GA	Population size	100
	Maximum number of generations	50
	Chromosomes length	83
	Mutation probability, $P_{m}$	1/83
	Crossover probability, $P_{c}$	0.6
	Elitism	Activated
	Precision, ɛ	0.05

### Simulations and model validation

The optimal values of $k,\theta ,a,b_{j=1,\ldots ,\;n}$ are presented in Table 4 and are different from theoretical values reported in the literature. We note that without AC, execution takes 97 s and consistency is achieved at the 34th generation. Thereafter, each chromosome in P(t) is assumed to be a CSP while each gene is considered as a variable. The global optimum is obtained at the 13th generation after generating the CSP at random and removing inconsistent values from the variable domains. The new execution takes 5 s. So, AC technique reduced execution time by 92 s. This gain also explains from 34 to 13 generations. As a result, GA needed fewer iterations to converge to a global optimum.

**Table 4:** Chromosome creation and parameter optimization.

Parameter	Modelled value		L−U	Genetic structure	Number of genes	Optimal value given by AC-GA
	GMC(1,n)	OGMC(1,n)				
Kerosene model						
θ	0.51	0.48	0.01–0.99	111	3	0.49
k	4	4	4–10	1111	4	4
−a	0.05	0.08	0.01–0.99	111,111	6	0.05
$b_{1}$	34.1	29.6	0–40	1,111,111,111	10	31.8
$b_{2}$	38.7	30.3	0–45	1,111,111,111	10	35.8
$b_{3}$	27.4	23.4	0–36	1,111,111,111	10	25.1
$b_{4}$	51.3	48.3	0–59	1,111,111,111	10	49.1
$b_{5}$	53.7	48.7	0–55	1,111,111,111	10	50.1
$b_{6}$	18.7	14.9	0–20	1,111,111,111	10	16.9
$b_{7}$	23.4	22.2	0–25	1,111,111,111	10	23.7
			Length of chromosome :		83	
LPG model						
θ	0.47	0.45	0.01–0.99	111	3	0.51
k	4	5	4–12	1111	4	5
−a	0.03	0.02	0.01–0.99	111,111	6	0.08
$b_{1}$	24.2	30.1	0–31	1,111,111,111	10	27.4
$b_{2}$	20.7	33.5	0–35	1,111,111,111	10	25.5
$b_{3}$	39.3	31.7	0–41	1,111,111,111	10	36.2
$b_{4}$	43.1	39.2	0–45	1,111,111,111	10	39.1
$b_{5}$	41.5	39.3	0–42	1,111,111,111	10	35.2
$b_{6}$	30.8	28.2	0–32	1,111,111,111	10	25.1
$b_{7}$	49.6	41.2	0–51	1,111,111,111	10	49.3
			Length of chromosome :		83	

**Fig. 2:**
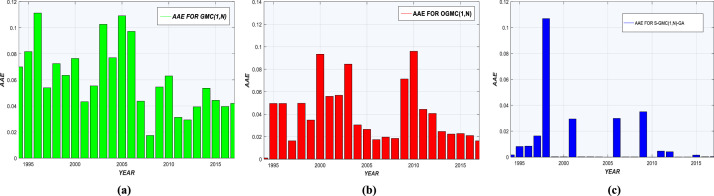
AAEs of (a) GMC, (b) OGMC, (c) S-GMC(1,n)-GA model for LPG training and test data.

**Fig. 3:**
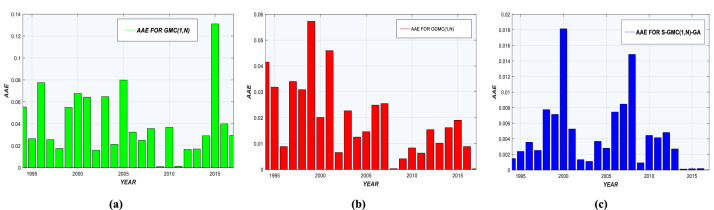
AAEs of (a) GMC, (b) OGMC, (c) S-GMC(1,n)-GA model for kerosene training and test data.

**Fig. 4:**
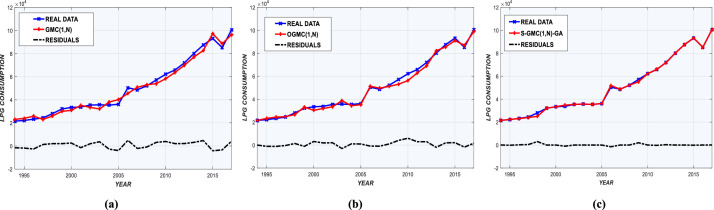
Observed-predicted LPG consumption and residuals for (a) GMC, (b) OGMC, (c) S-GMC(1,n)-GA.

**Fig. 5:**
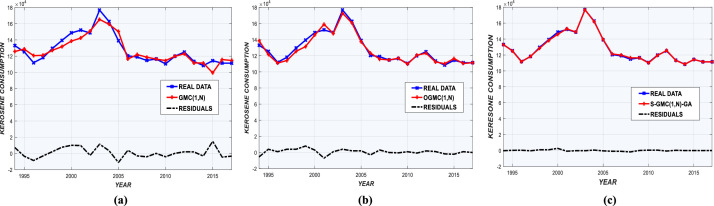
Observed-predicted kerosene consumption and residuals for (a) GMC, (b) OGMC, (c) S-GMC(1,n)-GA.

AAEs for each model are shown in Figs. 2(a-c) and 3(a-c). Prediction deviations given by Sequential-GMC(1,n)-GA are insignificant compared to GMC(1,n) and OGMC(1,n). Graphs of real and predicted consumption are shown in Fig. 4, Fig. 5, with annual residual curves. Forecast curves produced by Sequential-GMC(1,n)-GA almost fits to perfection with real data. Moreover, annual residuals curves of Sequential-GMC(1,n)-GA are much more spread over time than GMC(1,n) and OGMC(1,n). Therefore, Figs. 4 and 5 confirm the results given by Figs. 2(a-c) and 3(a-c).

Performance statistics are presented in Tables 5 and 6. According to these tables, Sequential-GMC(1,n)-GA significantly outperforms OGMC(1,n) and GMC(1,n) regarding all indicators, especially for MAPE. Figs. 2(a-c) and 3(a-c) confirm the superiority of Sequential-GMC(1,n)-GA on GMC(1,n) and OGMC(1,n).

**Table 5:** Performance of GMC(1,n), OGMC(1,n) and S-GMC(1,n)-GA for annual LPG demand prediction.

Measure	GMC(1,n)		OGMC(1,n)		S-GMC(1,n)-GA	
	Train	Test	Train	Test	Train	Test
*MAPE (%)*	7.1689	4.5205	6.0276	4.9891	1.3445	0.727
*r*	0.9947	0.9979	0.998	0.9975	0.9993	0.9999
*R^2^_adj_*	0.9772	0.9834	0.9761	0.972	0.9828	0.9916
*RMSE*	2.588	3.309	0.157	3.5821	0.8103	0.8325
*AAE*	0.2368	0.127	0.1336	0.1321	0.422	0.172

**Table 6:** Performance of GMC(1,n), OGMC(1,n) and S-GMC(1,n)-GA for annual kerosene demand prediction.

Measure	GMC(1,n)		OGMC(1,n)		S-GMC(1,n)-GA	
	Train	Test	Train	Test	Train	Test
*MAPE (%)*	6.9332	4.0314	4.9014	1.9429	1.6147	0.4396
*r*	0.9861	0.9975	0.9875	0.9994	0.9988	1.0000
*R^2^_adj_*	0.9354	0.9712	0.9365	0.9725	0.9610	0.9997
*RMSE*	4.3026	6.4998	1.9364	3.2911	8.273	8.308
*AAE*	0.2792	0.1534	0.1628	0.1204	0.6583	0.135

Table 7 allows comparing between Sequential-GMC(1,n)-GA and other hybrid GM based models. On the basis of MAPE, RMSE and AAE, we can conclude that Sequential-GMC(1,n)-GA offers better precision and much more reliable forecasting capabilities. Sequential-GMC(1,n)-GA achieves these outstanding results by accounting for all stimuli of PP consumption, all of which are a priori defined by a significant link with demand.

**Table 7:** Accuracy of hybrid GM-based models.

Author	Model	MAPE(%)	RMSE	AAE
Ofosu-Adarkwa et al. [13]	Verhulst-GM(1,n)	2.71	34	53
Lee and Tong [14]	GMGP(1,1)	4.20	–	–
Bahrami et al. [15]	Wavelet-GMPSO(1,5)	1.82	–	–
Zhao and Guo [16]	Rolling-ALO-GM(1,1)	4.04	–	–
Guefano et al. [17]	GM(1,1)-VAR(1)	1.63	15.42	1.5
This study	Sequential-GMC(1,n)-GA	1.44	0.8325	0.1204

Further tests and comparisons show that Sequential-GMC(1,n)-GA can perform as well as some new expert systems. Table 8 shows that Sequential-GMC(1,n)-GA can compete with VMD-EELM (Variational Mode Decomposition-Evolutionary Extreme Learning Machine) [21], VMDSVM-PSO (VMD coupled to SVM and improved by PSO) [22], and VMD-SRSVRCBCS (VMD hybridized with Self recurring mechanism and SVR, and optimized by CBCS) [23].

**Table 8:** Comparison between Sequential-GMC(1,n)-GA and other hybrid models, including artificial intelligence models.

Author	Model	MAPE(%)	RMSE	AAE
Yu et al. [18]	PSO-GA	0.54	–	–
Yu et al. [19]	EMD-RLSTM-ELM	3.56	–	–
Saxena et al. [20]	ARIMA-LR-ANN	2.45	–	–
Niu et al. [21]	VMD-EELM	2.96	471.781	–
Feng et al. [22]	VMD-SVM-PSO	–	3648.830	–
Zhang et al. [23]	VMD-SRSVRCBCS	0.9	–	62.3
This study	Sequential-GMC(1,n)-GA	1.44	0.8325	0.1204

### Convergence and stability tests

**Fig. 6:**
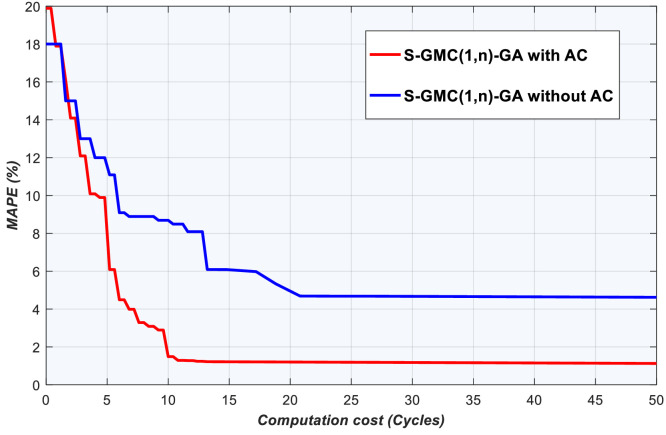
Convergence of S-GMC(1,n)-GA model with and without AC using LPG data.

**Fig. 7:**
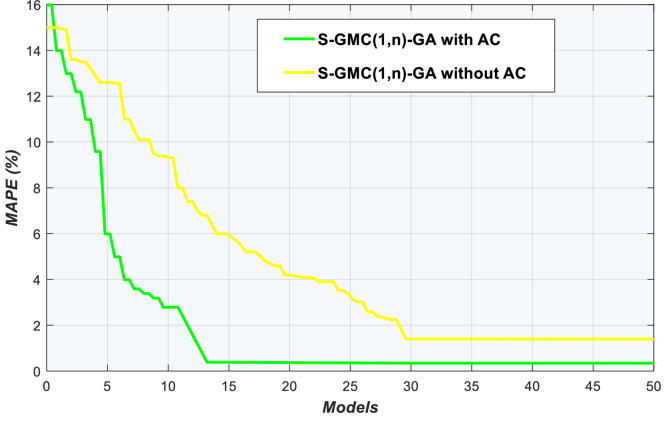
Convergence of S-GMC(1,n)-GA model with and without AC using kerosene data.

**Fig. 8:**
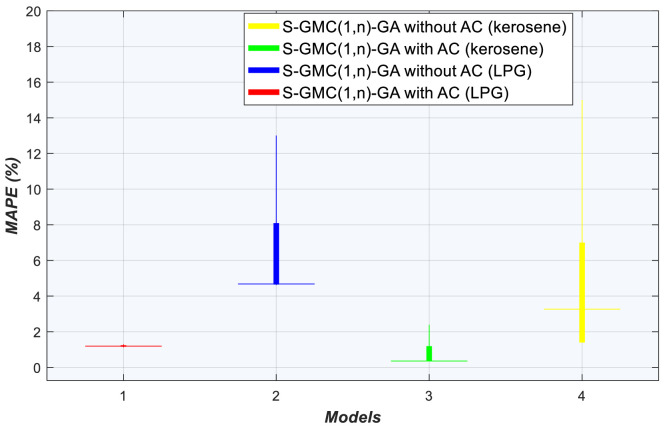
Boxplot of minimum MAPE after 50 evaluations from 10 runs.

We ran 10 trials of Sequential-GMC(1,n)-GA with and without AC, and the average value and standard deviation was computed to compare the speed of convergence and stability of the method (see Fig. 6, Fig. 7, Fig. 8).

Using LPG data, after 50 evaluations of Sequential-GMC(1,n), GA without AC finds mean solution at the 21st evaluation with MAPE of 4.622%. Using AC techniques combined with GA, mean solution is achieved at the 11th generation with MAPE of 1.178% (Fig. 6). Sequential-GMC(1,n)-GA without AC exhibit early convergence, and Sequential-GMC(1,n)-GA with AC catches up at around 7 evaluations. This better converging algorithm also display high stability (see Fig. 8).

With kerosene data, after 50 evaluations, Sequential-GMC(1,n)-GA without AC finds a solution at the 30th evaluation with MAPE of 1.4% whereas Sequential-GMC(1,n)-GA with AC technique finds a solution at the 13th evaluation with 0.388% MAPE (see Fig. 7). As for stability, Fig. 8 reveals a large difference between Sequential-GMC(1,n)-GA with AC and Sequential-GMC(1,n)-GA without AC. It is already well known that GA slows down its convergence speed if it needs to fine-tune weights and biases. However, there are two possible explanations why Sequential-GMC(1,n)-GA with AC outperforms Sequential-GMC(1,n)-GA without AC. First, the threshold to stop GA without AC is too low for this task so that efficiency was lost when a plateau is encountered. The second reason is that Sequential-GMC(1,n)-GA with AC is faster even in the initial search of near optimal solutions.

## Conclusion

In this paper, we proposed a Sequential-GMC(1,n)-GA hybrid model to forecast households’ PP consumption. Forecasting models were produced with training datasets from 1992 to 2008, while validation datasets include data for the period 2009–2017. The results show that Sequential-GMC(1,n)-GA hybrid model is suit to predict energy demand than competing models. This superiority results from two facts: first, instead of using theoretical values of θ and k, all parameters $\left(\theta ,\;k,\;a,\;b_{j=1,\ldots ,n}\right)$ are determined experimentally and optimized before each forecasting period. Second, the use of a sequential method and AC gathers the most recent data characteristics, reducing forecasting errors and CPU execution time. As a result, this study demonstrates that GM's performance can still be improved. Overall, the model has good adaptability and feasibility, and can extract a grey system's evolution law.

For forecasting purposes, the proposed model can be chosen out of other competing models provided the time series do not have a high degree of periodicity, volatility or are free from price shocks. This is because the Sequential-GMC(1,n)-GA will be unable to completely extract the evolution law due to the presence of disturbing characteristics. However, this issue can be solved by including a term that accounts for nonlinearities in a time-varying GMC(1,n) or by performing data preprocessing.

## Funding

This research did not receive any specific grant from funding agencies in the public, commercial, or not-for-profit sectors.

## CRediT authorship contribution statement

**Flavian Emmanuel Sapnken:** Conceptualization, Methodology, Software, Writing – original draft. **Ahmat Khazali Acyl:** Validation, Data curation. **Michel Boukar:** Visualization, Investigation. **Serge Luc Biobiongono Nyobe:** Software. **Jean Gaston Tamba:** Supervision, Validation, Writing – review & editing.

## Declaration of Competing Interest

The authors declare that they have no known competing financial interests or personal relationships that could have appeared to influence the work reported in this paper.

## Data Availability

Data will be made available on request.
